# Sequential HAIC-TACE combined with targeted and immunotherapy in hepatocellular carcinoma: a quality improvement study

**DOI:** 10.1097/JS9.0000000000002890

**Published:** 2025-07-02

**Authors:** Juncheng Wan, Bufu Tang, Wen Zhang, Yongjie Zhou, Chaoqiao Jin, Caihong Yu, Changyu Li, Wei Zhang, Zhuoyang Fan, Guowei Yang, Xudong Qu

**Affiliations:** a Department of Interventional Radiology, Zhongshan Hospital, Fudan University, Shanghai, China; b Shanghai Institute of Medical Imaging, Fudan University, Shanghai, China; c National Clinical Research Center of Interventional Medicine, Zhongshan Hospital, Fudan University, Shanghai, China

**Keywords:** hepatic artery infusion chemotherapy, hepatocellular carcinoma, immunotherapy, targeted therapy, transarterial chemoembolization

## Abstract

**Background:**

Hepatocellular carcinoma (HCC) remains a leading cause of cancer mortality. Emerging evidence suggests that quadruple therapy – combining hepatic artery infusion chemotherapy (HAIC), transcatheter arterial chemoembolization (TACE), targeted therapy, and immunotherapy – improves outcomes. However, few studies have directly compared this regimen with triple therapy (TACE plus targeted therapy and immunotherapy), and the optimal sequence of locoregional treatments within this regimen remains unclear.

**Objective:**

To compare the efficacy and safety of HAIC-first quadruple therapy, TACE-first quadruple therapy, and triple therapy in patients with unresectable HCC (uHCC).

**Materials and Methods:**

We retrospectively analyzed 158 patients with uHCC who received: HAIC-first quadruple therapy (HTTI, *n* = 61), TACE-first quadruple therapy (THTI, *n* = 41), or triple therapy (TTI, *n* = 56). Tumor response was assessed with modified RECIST; progression-free survival (PFS), overall survival (OS), and safety were compared.

**Results:**

The HTTI group achieved the highest objective response rate (ORR, 81.97%) and disease control rate (DCR, 96.72%), outperforming the THTI (ORR 73.17%, DCR 95.12%) and TTI groups (ORR 64.28%, DCR 85.71%). The median OS (mOS) was not reached in the HTTI group; the THTI group had a longer mOS (22.30 months) than the TTI group (19.00 months, *P* = 0.046). The median PFS (mPFS) was also longer in the HTTI group (12.90 months), compared to the THTI (10.40 months, P = 0.042) group and TTI groups (9.30 months, *P* < 0.001). Quadruple therapy increased grade 1 and 2 hypoalbuminemia, thrombocytopenia, and hand-foot syndrome, but grade 3 and 4 toxicities were similar across groups.

**Conclusion:**

Quadruple therapy demonstrates superior efficacy compared to triple therapy for uHCC. Initiating treatment with HAIC followed by TACE appears more effective than the reverse sequence and merits prospective validation.

## Introduction

Primary liver cancer is one of the most prevalent malignant tumors in China, with high incidence and mortality rates^[1]^. Hepatocellular carcinoma (HCC) accounts for 75–85% of these cases. Globally, despite a decline in hepatitis B and C infections, which has reduced liver cancer incidence in some countries, liver cancer remains the third leading cause of cancer-related death and the sixth most commonly diagnosed cancer as of 2022^[2]^. Due to its asymptomatic nature in the early stages, over 70% of patients are diagnosed at intermediate or advanced stages^[3]^ when curative surgical options are no longer feasible.

Transcatheter arterial chemoembolization (TACE) is a widely used non-surgical treatment^[4]^ and plays a crucial role in managing Barcelona Clinic Liver Cancer (BCLC) stage B HCC patients^[5]^. Systemic antitumor therapy, including targeted therapy and immunotherapy, is frequently incorporated into first-line treatment regimens such as atezolizumab combined with bevacizumab^[6]^, sorafenib^[7]^, and lenvatinib^[8]^. In recent years, numerous studies have highlighted the advantages of combining TACE with systemic therapy in HCC patients^[9–12]^, demonstrating benefits across various disease stages^[10,12]^. Although the BCLC guidelines^[5]^ have not yet been updated to reflect these findings, the latest Chinese guidelines have incorporated this approach^[13]^.

Recent studies^[14,15]^ have highlighted the significant role of hepatic artery infusion chemotherapy (HAIC) in treating unresectable HCC (uHCC). The commonly used FOLFOX regimen includes 5-fluorouracil, leucovorin, and oxaliplatin. HAIC involves direct infusion of chemotherapy drugs into hepatic artery branches supplying the tumor, achieving higher local drug concentrations in tumor cells compared to normal liver tissue. Building on this, combination therapies such as HAIC with TACE^[16]^, HAIC with targeted therapy and immunotherapy^[17]^, and quadruple therapy (HAIC with TACE, targeted therapy, and immunotherapy)^[18]^ have shown promising efficacy in uHCC. While some studies have demonstrated the benefits of quadruple therapy^[18–20]^, few have directly compared it with triple therapy (TACE plus targeted therapy and immunotherapy)^[21]^. Additionally, previous studies utilizing HAIC combined with TACE as locoregional treatment have predominantly administered TACE first. However, TACE embolizes tumor-supplying blood vessels, potentially hindering HAIC drug delivery and reducing treatment efficacy. We hypothesize that initiating treatment with HAIC, followed by TACE may be more advantageous. Therefore, in this study, we aimed to evaluate whether initiating treatment with HAIC-first followed by TACE would enhance efficacy compared to the TACE-first approach or triple therapy. In addition, to ensure transparency in the use of artificial intelligence tools during the preparation of this manuscript, we adhered to the TITAN (Transparency In The reporting of Artificial INtelligence) 2025 guidelines^[22]^.

## Materials and methods

### Patients

This single-center retrospective study was conducted in accordance with the Declaration of Helsinki and approved by the Institutional Ethics Committee. Informed consent for data use in research was obtained from all patients. We reviewed the records of 763 patients with intermediate or advanced HCC who underwent TACE or TACE combined with HAIC, targeted therapy, and immunotherapy between August 2018 and August 2024. Participants were selected based on specific inclusion and exclusion criteria.

### Study design and reporting standards

This study was a single-center retrospective review, reported in line with the Standards for Quality Improvement Reporting Excellence (SQUIRE) criteria^[23]^ to ensure transparency and consistency. The SQUIRE guidelines structured our methods, results, and discussion.

**Inclusion Criteria**:

- Intermediate or advanced-stage HCC;
- Age 18–90 years;
- Child–Pugh A or B liver function;
- Eastern Cooperative Oncology Group (ECOG) performance status ≤2;
- Complete follow-up data.

**Exclusion Criteria**:

- Previous locoregional therapies (e.g., ablation, TACE, or radiotherapy);
- Absence of measurable lesions at baseline as per the modified RECIST (mRECIST) 1.1 criteria^[24]^;
- History of other malignancies;
- Tumor with diffuse or poorly defined margins;
- Life expectancy <6 months;
- Non-HCC pathology.

**HIGHLIGHTS**

- This quality improvement study evaluates the sequential combination of HAIC, TACE, targeted therapy, and immunotherapy for treating unresectable hepatocellular carcinoma (HCC), with strong clinical applicability.
- The HTTI treatment sequence (HAIC followed by TACE) demonstrates superior efficacy, with the highest objective response rate (81.97%) and disease control rate (96.72%). The HTTI group shows a significantly longer progression-free survival (12.90 months), with adverse reactions remaining within an acceptable range. The study emphasizes improvements in treatment sequencing to optimize outcomes for advanced HCC patients.
- Additionally, this study compares the quadruple therapy regimen (HAIC, TACE, targeted therapy, and immunotherapy) with the commonly used TACE combined with targeted therapy and immunotherapy, providing more comprehensive evidence-based support for the quadruple therapy approach.
- The patient cohort in this study has notable clinical characteristics, including a high proportion of stage C patients (87.3%), portal vein tumor thrombosis (PVTT) patients (70.9%), VP3 and 4 type patients, and those meeting the Up-to-seven criteria (81.0%). Furthermore, 43.7% of patients had bilateral liver involvement. These patients are typically excluded from large clinical trials and often receive suboptimal results with single-agent targeted and immunotherapy as per international guidelines. This study offers a promising solution for these difficult-to-treat patients.

HCC diagnosis was confirmed by pathological results or American Association for the Study of Liver Diseases (AASLD) criteria^[25]^. For patients not meeting AASLD criteria (e.g., atypical features or non-HCC subtypes), diagnosis was established pathologically.

### Treatment protocols

All procedures were performed by physicians with over 15 years of experience in TACE procedures. Patients in all groups received the same TACE protocol.

### Triple therapy (TTI group)

Patients in the TTI group underwent triple therapy, consisting of TACE combined with targeted therapy and immunotherapy.

TACE were performed in a sterile digital subtraction angiography (DSA) room. Patients, typically supine, underwent puncture usually via the right femoral artery (left femoral or radial arteries if necessary). Routine oxygen, electrocardiographic monitoring, and intravenous access were established. Pain management and antiemetics were administered. Using the Seldinger technique, a catheter was advanced into the hepatic artery for angiography to evaluate the tumor blood supply. If needed, the catheter was advanced to the left gastric or subphrenic artery. Once positioned in the tumor-supplying artery, embolization was performed, followed by angiography to assess tumor staining adequacy. For large tumors with persistent staining, additional embolization was performed. Chemotherapeutic agents (epirubicin or doxorubicin, 30–60 mg) and embolizing agents (iodized oil ≤20 mL; microspheres or gelatin sponge if required) were used. Efforts were made to selectively embolize the target vessels, minimizing damage to normal liver tissue. Post-procedure care included pain management, liver protection, acid suppression, and antiemetics. Embolization syndrome was monitored and treated as needed.

### HAIC regimen

The quadruple therapy groups received the same HAIC regimen.

Chemotherapy was administered via catheter into the hepatic artery using the FOLFOX regimen: oxaliplatin 100–150 mg (infused over 2 h), calcium folinate 400 mg (infused over 2 h), and 5-fluorouracil 2.0–3.5 g (infused over 20 h). Intravenous antiemetics (e.g., tropisetron 5 mg or palonosetron 0.25 mg) were administered. If abdominal pain occurred, antispasmodics or analgesics were provided. For persistent pain, 1–2 mL of 1% lidocaine was slowly injected via microcatheter. If pain remained intolerable, the chemotherapy infusion was discontinued.

### HAIC-first quadruple therapy (HTTI group)

HAIC was performed first, immediately followed by TACE on the same day. After HAIC infusion, the patient returned to the DSA room for TACE (angiography and embolization according to the standard protocol). Postoperative care mirrored the TACE group.

### TACE-first quadruple therapy (THTI group)

TACE was performed first. Immediately after embolization, the catheter was retained in the tumor-feeding artery. The patient was transferred to the ward on the same day for HAIC chemotherapy. After chemotherapy, the catheter and sheath were removed, and compression applied. Postoperative care was identical to the TACE group.

### Systemic therapy

The targeted therapies used in this study included: apatinib (250 mg, once daily orally), anlotinib (12 mg, once daily orally), bevacizumab (5 mg/kg, once every 3 weeks intravenously), donafenib (200 mg, twice daily orally), lenvatinib (12 mg/day for weight ≥60 kg, 8 mg/day for <60 kg, once daily orally), regorafenib (160 mg, once daily orally), and sorafenib (400 mg, twice daily orally).

The immunotherapy drugs used in this study included atezolizumab (1200 mg, once every 3 weeks intravenously), durvalumab + tremelimumab (durvalumab: 1500 mg, once every 4 weeks intravenously; tremelimumab: 300 mg, once every 4 weeks intravenously), camrelizumab (200 mg, once every 3 weeks intravenously), pembrolizumab (200 mg, once every 3 weeks intravenously), toripalimab (240 mg, once every 3 weeks intravenously), tislelizumab (200 mg, once every 3 weeks intravenously), and sintilimab (200 mg, once every 3 weeks intravenously) (Supplementary Digital Content Table 1, available at, http://links.lww.com/JS9/E549).

After locoregional therapy (TACE in the TTI/HTTI groups, HAIC in the THTI group), patients initiated oral targeted therapy on day 1, continuing until disease progression or severe toxicity. Immunotherapy infusions were scheduled based on the first infusion date, with bevacizumab and atezolizumab administered on the same day when applicable.

The selection of targeted and immunotherapy drugs was determined based on factors such as drug prices, charitable donation programs, local insurance reimbursement policies, and patient preferences to ensure accessibility and individualized treatment approaches.

### Management of portal vein tumor thrombosis with portal hypertension

For patients with VP3 and 4 type tumor thrombosis causing portal vein obstruction and hypertension, additional interventions (portal vein stent placement, iodine-125 seed implantation) were performed alongside locoregional treatments to improve blood flow and efficacy (Supplementary Materials and Method 1, available at, http://links.lww.com/JS9/E549).

### Management of severe arteriovenous fistula

In patients with severe arteriovenous fistula (e.g., hepatic artery-hepatic vein, hepatic artery-portal vein) with blood flow away from the portal vein, pretreatment embolization (gelatin sponge, spring coils, or tissue glue) was performed prior to locoregional therapy to optimize efficacy. (Supplementary Materials and Method 2, available at, http://links.lww.com/JS9/E549).

### Tumor downstaging followed by surgical treatment (liver transplantation/liver resection)

The opportunity for surgical treatment after tumor downstaging was determined by a multidisciplinary team consisting of surgeons, interventional specialists, and anesthesiologists (Supplementary Materials and Method 3, available at, http://links.lww.com/JS9/E549).

### Efficacy evaluation

Tumor response was assessed 4–10 weeks post-treatment using enhanced computed tomography (CT) or magnetic resonance imaging (MRI). Two experienced radiologists evaluated radiological response per the modified Response Evaluation Criteria in Solid Tumors (mRECIST) 1.1 criteria^[24]^: complete response (CR) – disappearance of any intratumoral arterial enhancement; partial response (PR) –≥30% decrease in the sum of diameters of viable target lesions; stable disease (SD) – Neither sufficient shrinkage for PR nor sufficient increase for progressive disease (PD); PD – ≥20% increase in the sum of diameters of viable target lesions or appearance of new lesions. The objective response rate (ORR) was CR + PR. Disease control rate (DCR) was CR + PR + SD.

Portal vein tumor thrombus (PVTT) efficacy was evaluated was evaluated based on the criteria proposed by Zhou^[26]^ and Yoon^[27]^: CR – complete disappearance; PR – ≥ 50% reduction in thrombus diameter; SD – <50% reduction or <25% increase; PD – ≥25% increase.

Treatment-related adverse events (AEs) were evaluated using the Common Terminology Criteria for AEs (CTCAE) version 5.0. Standardized management strategies were applied for common AEs, including thrombocytopenia, hypoalbuminemia, and hand-foot syndrome, particularly with oxaliplatin use. Hand-foot syndrome was managed with dose adjustment. Hypoalbuminemia was treated with human serum albumin, while thrombocytopenia and leukopenia were managed with thrombopoietin and G-CSF respectively. Abdominal pain related to chemotherapy infusion or embolization was managed by slowing the infusion rate, adjusting the dose, and administering analgesics or antispasmodics. Dose modifications and treatment interruptions due to AEs were recorded throughout the treatment course. Treatment-related mortality was monitored during follow-up.

### Follow-up and repeat treatment

Patients received routine follow-up every 4–10 weeks, including medical history, physical examination, laboratory tests, chest CT, and dynamic enhanced abdominal CT/MRI. Other imaging was performed as needed. If tumor markers or imaging suggest tumor persistence, repeat treatment was considered after multidisciplinary discussion. Treatment was discontinued if nonprogression per mRECIST was not achieved after at least two consecutive interventional treatments. New liver tumors distant from the primary lesion were considered PD but not a contraindication for repeat treatment of both residual and new lesions.

Repeat treatment was discontinued if the primary tumor did not meet the treatment goals after more than two treatments and new lesions appeared, or if ECOG PS >2, persistent liver dysfunction, or intolerable toxic reactions. Subsequent treatment after PD was decided via multidisciplinary consultation.

### Statistical analysis

Continuous variables were compared using independent or paired sample *t*-tests; categorical variables with chi-square tests. In cases where multiple comparisons were made, the Bonferroni correction or false discovery rate was applied to control for type I error. Mann–Whitney *U* tests and chi-square tests were applied to compare clinical parameters between two groups. Progression-free survival (PFS) was the time from the first treatment to clinical progression or death; Overall survival (OS) was the time from the first treatment to death. Kaplan–Meier curves were used to analyze OS and PFS, with comparisons made using log-rank tests. Propensity score matching (PSM) analysis was used to reduce selection bias and balance the patient characteristics (Supplementary Materials and Method 4, available at, http://links.lww.com/JS9/E549). Univariate and multivariate Cox proportional hazards models were used to analyze factors associated with survival. Missing data were handled using multiple imputation or complete case analysis, depending on the nature and extent of the missing data. All tests were two-sided, and a *P*-value <0.05 was considered statistically significant. All analyses were conducted using SPSS 27.0 and R version 4.4.0.

## Results

### Patient characteristics

**Figure 1.**
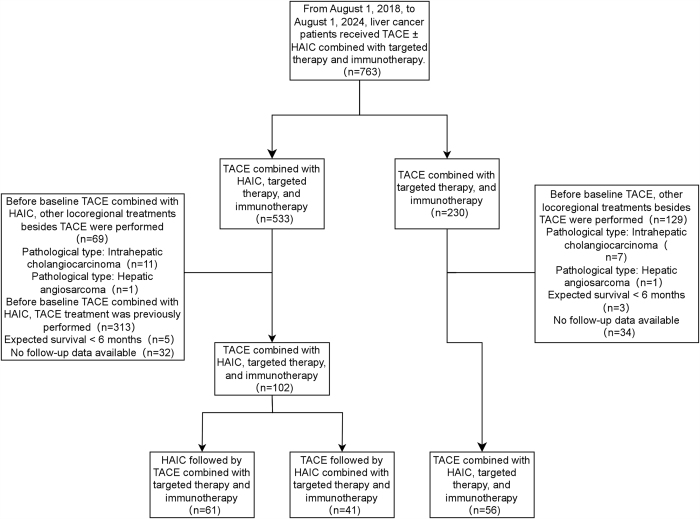
Patient enrollment and exclusion flowchart.

The clinical and imaging data are in Table 1. A total of 158 patients with uHCC were included: 61 in the HTTI group, 41 in the THTI group, and 56 in the TTI group (Fig. 1). Among them, 61 patients received HTTI, 41 THTI, and 56 TTI. The final follow-up was 15 February 2025; median follow-up was 16.25 months. HTTI patients received 193 cycles of HAIC-TACE (range: 1–8; median: 3), THTI patients received 137 cycles of TACE-HAIC (range: 1–9; median: 3), and TTI patients received 213 cycles of TACE (range: 1–9; median: 3.5).

**Table 1:** Baseline characteristics of patients

	HTTI group (*n* = 61)	THTI group (*n* = 41)	TTI group (*n* = 56)	Test value	*P* value	*P* value**
Age (years)				0.945	0.623	0.342
≥60	25 (40.98)	13 (31.71)	20 (35.71)			
<60	36 (59.02)	28 (68.29)	36 (64.29)			
Sex				7.935	0.019	0.154
Male	55 (90.16)	39 (95.12)	43 (76.79)			
Female	6 (9.84)	2 (4.88)	13 (23.21)			
BCLC stage				1.833	0.4	0.304
B	5 (8.20)	6 (14.63)	9 (16.07)			
C	56 (91.80)	35 (85.37)	47 (83.93)			
ECOG PS				0.641	0.726	0.987
0	52 (85.25)	35 (85.37)	45 (80.36)			
1	9 (14.75)	6 (14.63)	11 (19.64)			
PVTT				9.536	0.008	0.163
Present	45 (73.77)	35 (85.37)	32 (57.14)			
Absent	16 (26.23)	6 (14.63)	24 (42.86)			
HVTT				1.249	0.536	0.266
Present	26 (42.62)	13 (31.71)	21 (37.50)			
Absent	35 (57.38)	28 (68.29)	35 (62.50)			
IVTT				0.404	0.851	1.000
Present	7 (11.48)	5 (12.20)	5 (8.93)			
Absent	54 (88.52)	36 (87.80)	51 (91.07)			
Extrahepatic metastasis				34.491	<0.001	0.278
Present	45 (73.77)	34 (82.93)	17 (30.36)			
Absent	16 (26.23)	7 (17.07)	39 (69.64)			
Preoperative radical treatment				3.528	0.177	0.062
Present	8 (13.11)	1 (2.44)	6 (10.71)			
Absent	53 (86.89)	40 (97.56)	50 (89.29)			
Liver function Child–Pugh				5.593	0.061	0.253
B	9 (14.75)	3 (7.32)	14 (25.00)			
A	52 (85.25)	38 (92.68)	42 (75.00)			
Tumor type				4.576	0.101	0.979
Massive	43 (70.49)	29 (70.73)	30 (53.57)			
Nodular	18 (29.51)	12 (29.27)	26 (46.43)			
Tumor involvement				1.451	0.484	0.360
Hemiliver	25 (40.98)	16 (39.02)	28 (50.00)			
Both hemilivers	36 (59.02)	25 (60.98)	28 (50.00)			
Main tumor size (mm)	104.31 ± 46.30	97.85 ± 46.08	66.00 (39.75-117.75)	*H* = 8.848*	0.012	0.491
Tumor number				10.087	0.006	0.228
>3	36 (59.02)	29 (70.73)	22 (39.29)			
≤3	25 (40.98)	12 (29.27)	34 (60.71)			
Imaging cirrhosis				0.853	0.653	0.762
Present	39 (63.93)	25 (60.98)	39 (69.64)			
Absent	22 (36.07)	16 (39.02)	17 (30.36)			
Etiology				3.619	0.164	0.086
Hepatitis B or hepatitis C	45 (73.77)	36 (87.80)	47 (83.93)			
Non-hepatitis	16 (26.23)	5 (12.20)	9 (16.07)			

*Kruskal–Wallis *H* test.

**HTTI group vs THTI group.

Values are presented as *n* (%).

*P* values were calculated using a two-sided *χ*^2^ test.

BCLC: Barcelona Clinic Liver Cancer; HVTT: hepatic vein tumor thrombus; IVTT: inferior vena cava tumor thrombus; PVTT: portal vein tumor thrombus.

No significant differences in baseline characteristics between the HTTI and THTI groups (*P* > 0.05). However, significant differences were observed among the three groups for gender, PVTT, extrahepatic metastasis, and tumor number (*P* < 0.05). Specifically, the TTI group had a higher proportion of female patients, smaller primary tumor sizes, fewer tumors, and lower incidences of PVTT and extrahepatic metastasis. Therefore, we performed a PSM between the quadruple therapy group and the triple therapy group. After matching, the baseline characteristics between the two groups were well balanced (Supplementary Table 2, available at, http://links.lww.com/JS9/E549).

### Tumor response

**Figure 2.**
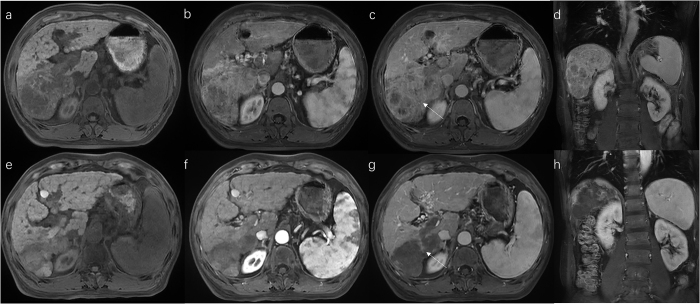
Pre- and post-HTTI treatment imaging of a patient. Pretreatment images: (A) T1-weighted image, (B) arterial phase T1-weighted image, (C) portal phase T1-weighted image, and (D) coronal view. Posttreatment images: (E) T1-weighted image, (F) arterial phase T1-weighted image, (G) portal phase T1-weighted image, and (H) coronal view.

The tumor response is shown in Table 2. According to the mRECIST, the HTTI group had the highest ORR (81.97%), DCR (96.72%), PR rate (73.77%), and the CR rate (8.20%). The ORR and DCR in the THTI group were slightly higher than those in the TTI group. Typical cases are presented in Figure 2.

(E) The tumor lesions exhibited a reduction in size, with evident necrotic areas, indicating a positive treatment response.

(F) The tumor lesions showed reduced enhancement, with slight peripheral enhancement, suggesting treatment response with residual activity at the margins.

(G) The portal vein tumor thrombus regressed, with partial restoration of normal blood flow in the right portal vein. The arrow indicates the tumor thrombus in the right branch of the portal vein.

**Table 2:** Treatment efficacy evaluated by mRECIST criteria

	HTTI group		THTI group		TTI group	
	mRECIST, *n* = 61		mRECIST, *n* = 41		mRECIST, *n* = 56	
Tumor response	*n*	%	*n*	%	*n*	%
CR	5	8.20	1	2.44	2	3.57
PR	45	73.77	29	70.73	34	60.71
SD	9	14.75	9	21.95	12	21.43
PD	2	3.28	2	4.88	8	14.29
ORR (CR + PR)	81.97% (50/61)		73.17% (30/41)		64.28% (36/56)	
DCR (CR + PR + SD)	96.72% (59/61)		95.12% (39/41)		85.71% (48/56)	

CR: complete response; DCR: disease control rate; ORR: objective response rate; PD: progressive disease; PR: partial response; SD: stable disease.

PVTT response is in Table 3. PVTT was present in 45/61 HTTI, 35/41 THTI, and 32/56 TTI patients. According to the criteria proposed by Zhou *et al* ^[26]^, in the HTTI group, the ORR, DCR and PR for PVTT were 62.22%, 97.78%, and 57.78%, respectively, comparable to those in the THTI group but higher than those in the TTI group. A representative case is shown in Figure 2.

**Table 3:** Response assessment of portal vein tumor thrombus

	HTTI group		THTI group		TTI group	
	mRECIST, *n* = 45		mRECIST, *n* = 35		mRECIST, *n* = 32	
Portal vein tumor thrombus response	*n*	%	*n*	%	*n*	%
CR	2	4.44	0	0	0	0
PR	26	57.78	22	62.86	5	15.63
SD	16	35.56	11	31.43	24	75
PD	1	2.22	2	5.71	3	9.38
ORR (CR + PR)	62.22% (28/45)		62.86% (22/35)		15.63% (5/32)	
DCR (CR + PR + SD)	97.78% (44/45)		94.29% (33/35)		90.63% (29/32)	

CR: complete response; DCR: disease control rate; ORR: objective response rate; PD: progressive disease; PR: partial response; SD: stable disease.

### Survival outcomes

**Figure 3.**
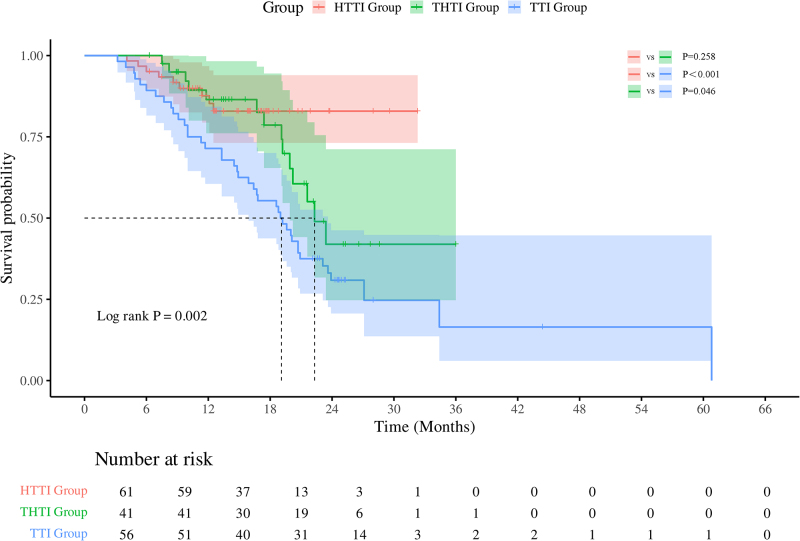
Kaplan–Meier analysis of overall survival among different groups.

Median OS (mOS) in the HTTI group was not reached. In the THTI group, the mOS was 22.30 months (95% CI: 19.39–25.22), significantly longer than the the TTI group’s 19.00 months (95% CI: 15.09–22.91; *P* = 0.046) (Fig. 3).

**Figure 4.**
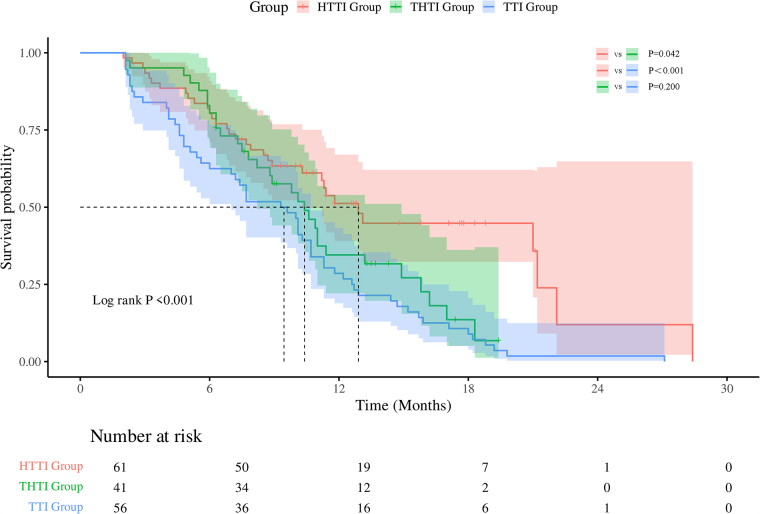
Kaplan–Meier analysis of progression-free survival among different groups.

mPFS in the HTTI group was 12.90 months (95% CI: 10.65–15.15), significantly longer than in the THTI group (10.40 months, 95% CI: 8.19–12.61; *P* = 0.042). The TTI group had the shortest PFS (9.30 months, 95% CI: 7.10–11.50; *P* < 0.001) (Fig. 4).

Univariate Cox analysis for OS identified quadruple therapy, age ≥60 years, and extrahepatic metastasis as protective factors, while BCLC stage C, PVTT, Child–Pugh class B, and elevated direct bilirubin levels were associated with higher risk. Multivariate analysis identified quadruple therapy as an independent protective factor for OS; elevated direct bilirubin remained an independent high-risk factor (Table 4).

**Table 4:** Univariate and multivariate Cox regression analyses of the prognostic factors for OS

Variables	Group	*n*	OS univariate			OS multivariate*	
			HR (95% CI)	*P* value		HR (95% CI)	*P* value
**Treatment**	**Quadruple Therapy**	102	1.000 (Reference)			1.000 (Reference)	
	**Triple Therapy**	56	2.371 (1.408–3.991)	0.001		2.608 (1.314–5.178)	0.006
**Sex**	**Female**	21	1.000 (Reference)				
	**Male**	137	0.666 (0.358–1.237)	0.198			
**Age (years)**	**<60**	100	1.000 (Reference)			1.000 (Reference)	
	**≥60**	58	0.578 (0.334–0.999)	0.05		0.775 (0.434–1.385)	0.389
**BCLC stage**	**B**	20	1.000 (Reference)			1.000 (Reference)	
	**C**	138	2.683 (1.141–6.309)	0.024		1.711 (0.588–4.983)	0.324
**ECOG PS**	**0**	132	1.000 (Reference)				
	**1**	26	1.104 (0.575–2.121)	0.766			
**PVTT**	**Absent**	46	1.000 (Reference)			1.000 (Reference)	
	**Present**	112	1.946 (1.103–3.435)	0.022		1.842 (0.881–3.853)	0.105
**HVTT**	**Absent**	98	1.000 (Reference)				
	**Present**	60	1.105 (0.661–1.848)	0.703			
**IVTT**	**Absent**	141	1.000 (Reference)				
	**Present**	17	1.573 (0.746–3.317)	0.234			
**Extrahepatic metastasis**	**Absent**	62	1.000 (Reference)			1.000 (Reference)	
	**Present**	96	0.580 (0.353–0.952)	0.031		0.873 (0.459–1.661)	0.678
**Preoperative radical treatment**	**Absent**	143	1.000 (Reference)				
	**Present**	15	0.958 (0.435–2.110)	0.916			
**Child–Pugh class**	**A**	132	1.000 (Reference)			1.000 (Reference)	
	**B**	26	1.988 (1.124–3.515)	0.018		1.480 (0.803–2.727)	0.209
**Tumor type**	**Massive**	102	1.000 (Reference)				
	**Nodular**	56	1.288 (0.782–2.119)	0.32			
**Tumor involvement**	**Both hemilivers**	89	1.000 (Reference)				
	**Hemiliver**	69	0.680 (0.406–1.140)	0.143			
**Main tumor size (mm)**	**<100**	89	1.000 (Reference)				
	**≥100**	69	1.403 (0.846–2.326)	0.189			
**Tumor number**	**>3**	87	1.000 (Reference)				
	**≤3**	71	1.240 (0.755–2.034)	0.395			
**Up-to-seven**	**>7**	128	1.000 (Reference)				
	**≤7**	30	1.024 (0.578–1.814)	0.935			
**Imaging cirrhosis**	**Absent**	55	1.000 (Reference)				
	**Present**	103	1.104 (0.644–1.894)	0.718			
**Hepatitis condition**	**Hepatitis B or hepatitis C**	128	1.000 (Reference)				
	**Non-hepatitis**	30	0.975 (0.508–1.871)	0.94			
**Severe arteriovenous fistula**	**Absent**	142	1.000 (Reference)				
	**Present**	16	0.476 (0.173–1.313)	0.152			
**Portal vein stenting combined with internal radiation therapy**	**Combined**	12	1.000 (Reference)				
	**Not combined**	146	1.675 (0.523–5.363)	0.385			
**TBIL (μmol/L)**	**>17.1**	52	1.000 (Reference)				
	**≤17.1**	106	0.731 (0.430–1.241)	0.245			
**DBIL (μmol/L)**	**>6.8**	51	1.000 (Reference)			1.000 (Reference)	
	**≤6.8**	107	0.540 (0.321–0.910)	0.021		0.566 (0.333–0.962)	0.035
**TP (g/L)**	**<60**	8	1.000 (Reference)				
	**≥60**	150	0.690 (0.250–1.906)	0.474			
**ALB (g/L)**	**<35**	25	1.000 (Reference)				
	**≥35**	133	0.695 (0.361–1.337)	0.276			
**ALT (U/L)**	**>40**	76	1.000 (Reference)				
	**≤40**	82	0.907 (0.550–1.497)	0.704			
**AST (U/L)**	**>40**	105	1.000 (Reference)				
	**≤40**	53	0.807 (0.478–1.364)	0.424			
**AFP (ng/mL)**	**>400**	32	1.000 (Reference)				
	**≤400**	126	1.116 (0.594–2.096)	0.732			
**PIVKA-II(ng/mL)**	**>1000**	106	1.000 (Reference)				
	**≤1000**	52	0.780 (0.463–1.315)	0.351			

* Multivariate Cox regression analysis was performed using the forward stepwise method in SPSS.

AFP: alpha-fetoprotein; ALB: albumin; ALT: alanine aminotransferase; AST: aspartate aminotransferase; BCLC: Barcelona Clinic Liver Cancer; CI: confidence interval; DBIL: direct bilirubin; HAIC: hepatic arterial infusion chemotherapy; HR: hazard ratio; HVTT: hepatic vein tumor thrombus; IVTT: inferior vena cava tumor thrombus; PIVKA-II: protein induced by vitamin K absence or antagonist-II; PVTT: portal vein tumor thrombus; TACE: transcatheter arterial chemoembolization; TBIL: total bilirubin; TP: total protein.

For PFS, univariate Cox analysis indicated quadruple therapy, male, and age ≥60 years as protective factors, while BCLC stage C, PVTT, Child–Pugh class B, and bilateral tumor involvement were high-risk factors. Multivariate analysis confirmed quadruple therapy as a protective factor; bilateral tumor involvement was a high-risk factor (Table 5).

**Table 5:** Univariate and multivariate Cox regression analyses of the prognostic factors for PFS

Variables	Group	n	OS univariate			OS multivariate*	
			HR (95% CI)	*P* value		HR (95% CI)	*P* value
**Treatment**	**Quadruple therapy**	102	1.000 (Reference)			1.000 (Reference)	
	**Triple therapy**	56	1.849 (1.289–2.652)	<.001		2.140 (1.466–3.123)	<.001
**Sex**	**Female**	21	1.000 (Reference)			1.000 (Reference)	
	**Male**	137	0.508 (0.309–0.836)	0.008		0.590 (0.344–1.012)	0.055
**Age (years)**	**<60**	100	1.000 (Reference)			1.000 (Reference)	
	**≥60**	58	0.560 (0.375–0.837)	0.005		0.667 (0.440–1.011)	0.057
**BCLC stage**	**B**	20	1.000 (Reference)			1.000 (Reference)	
	**C**	138	1.801 (1.004–3.230)	0.049		1.308 (0.636–2.692)	0.466
**ECOG PS**	**0**	132	1.000 (Reference)				
	**1**	26	1.160 (0.728–1.848)	0.532			
**PVTT**	**Absent**	46	1.000 (Reference)			1.000 (Reference)	
	**Present**	112	1.511 (1.009–2.263)	0.045		1.533 (0.912–2.576)	0.107
**HVTT**	**Absent**	98	1.000 (Reference)				
	**Present**	60	1.016 (0.699–1.478)	0.932			
**IVTT**	**Absent**	141	1.000 (Reference)				
	**Present**	17	1.444 (0.808–2.579)	0.215			
**Extrahepatic metastasis**	**Absent**	62	1.000 (Reference)				
	**Present**	96	0.823 (0.567–1.195)	0.307			
**Preoperative radical treatment**	**Absent**	143	1.000 (Reference)				
	**Present**	15	0.591 (0.310–1.127)	0.110			
**Child–Pugh class**	**A**	132	1.000 (Reference)			1.000 (Reference)	
	**B**	26	1.807 (1.144–2.856)	0.011		1.393 (0.854–2.274)	0.184
**Tumor type**	**Massive**	102	1.000 (Reference)				
	**Nodular**	56	1.033 (0.710–1.501)	0.866			
**Tumor involvement**	**Both hemilivers**	89	1.000 (Reference)			1.000 (Reference)	
	**Hemiliver**	69	0.589 (0.404–0.858)	0.006		0.572 (0.387–0.846)	0.005
**Main tumor size (mm)**	**<100**	89	1.000 (Reference)				
	**≥100**	69	1.021 (0.706–1.475)	0.914			
**Tumor number**	**>3**	87	1.000 (Reference)				
	**≤3**	71	0.806 (0.560–1.158)	0.243			
**Up-to-seven**	**>7**	128	1.000 (Reference)				
	**≤7**	30	0.998 (0.648–1.538)	0.993			
**Imaging cirrhosis**	**Absent**	55	1.000 (Reference)				
	**Present**	103	1.124 (0.765–1.650)	0.551			
**Hepatitis condition**	**Hepatitis B or hepatitis C**	128	1.000 (Reference)				
	**Non-hepatitis**	30	0.698 (0.431–1.132)	0.145			
**Severe arteriovenous fistula**	**Absent**	142	1.000 (Reference)				
	**Present**	16	0.667 (0.361–1.234)	0.197			
**Portal vein stenting combined with internal radiation therapy**	**Combined**	12	1.000 (Reference)				
	**Not Combined**	146	1.012 (0.491–2.085)	0.974			
**TBIL (μmol/L)**	**>17.1**	52	1.000 (Reference)				
	**≤17.1**	106	1.385 (0.926–2.073)	0.113			
**DBIL (μmol/L)**	**>6.8**	51	1.000 (Reference)				
	**≤6.8**	107	1.120 (0.750–1.672)	0.580			
**TP (g/L)**	**<60**	8	1.000 (Reference)				
	**≥60**	150	0.721 (0.316–1.645)	0.437			
**ALB (g/L)**	**<35**	25	1.000 (Reference)				
	**≥35**	133	0.906 (0.547–1.501)	0.703			
**ALT (U/L)**	**>40**	76	1.000 (Reference)				
	**≤40**	82	1.216 (0.847–1.747)	0.289			
**AST (U/L)**	**>40**	105	1.000 (Reference)				
	**≤40**	53	0.992 (0.681–1.444)	0.966			
**AFP (ng/mL)**	**>400**	32	1.000 (Reference)				
	**≤400**	126	0.980 (0.620–1.549)	0.932			
**PIVKA-II (ng/mL)**	**>1000**	106	1.000 (Reference)				
	**≤1000**	52	1.055 (0.726–1.534)	0.779			

* Multivariate Cox regression analysis was performed using the forward stepwise method in SPSS.

ALB: albumin; ALT: alanine aminotransferase; AST: aspartate aminotransferase; AFP: alpha-fetoprotein; BCLC: Barcelona Clinic Liver Cancer; CI: confidence interval; DBIL: direct bilirubin; HAIC: hepatic arterial infusion chemotherapy; HR: hazard ratio; HVTT: hepatic vein tumor thrombus; IVTT: inferior vena cava tumor thrombus; PIVKA-II: protein induced by vitamin K absence or antagonist-II; PVTT: portal vein tumor thrombus; TACE: transcatheter arterial chemoembolization; TBIL: total bilirubin; TP: total protein.

### Safety

Since the HTTI and THTI groups used the same drugs and embolization agents, they were combined into the quadruple therapy group for AEs analysis (Table 6). The quadruple therapy group showed a higher frequency of hypoalbuminemia, thrombocytopenia, and hand-foot syndrome (*P* < 0.05). However, no significant difference in grade 3 and 4 AE frequency was found.

**Table 6:** Treatment-related adverse events

Adverse event	All grades				Grade 3–4 adverse events		
	Quadruple therapy group (*n* = 102)	Triple therapy group (*n* = 56)	*P* value		Quadruple therapy group (*n* = 102)	Triple therapy group (*n* = 56)	*P* value
**Hyperbilirubinemia**	9 (8.82)	2 (3.57)	0.330		0 (0.00)	0 (0.00)	
**Hypoalbuminemia**	36 (35.29)	7 (12.50)	0.002		0 (0.00)	0 (0.00)	
**Elevated ALT**	8 (7.84)	4 (7.14)	1.000		2 (1.96)	2 (3.57)	0.615
**Elevated AST**	14 (13.73)	4 (7.14)	0.213		1 (0.98)	2 (3.57)	0.287
**Elevated creatinine**	3 (2.94)	0 (0.00)	0.553		0 (0.00)	0 (0.00)	
**Thrombocytopenia**	23 (22.55)	23 (41.07)	0.014		4 (3.92)	3 (5.36)	0.699
**Leukopenia**	30 (29.41)	19 (33.93)	0.557		0 (0.00)	1 (1.79)	0.354
**Anemia**	35 (34.31)	17 (30.36)	0.613		3 (2.94)	2 (3.57)	1.000
**Diarrhea**	5 (4.90)	6 (10.71)	0.199		0 (0.00)	0 (0.00)	
**Ulceration**	3 (2.94)	3 (5.36)	0.667		0 (0.00)	0 (0.00)	
**Fever**	43 (42.16)	19 (33.93)	0.311		0 (0.00)	0 (0.00)	
**Rash**	2 (1.96)	1 (1.79)	1.000		0 (0.00)	0 (0.00)	
**Nausea**	25 (24.51)	8 (14.29)	0.130		0 (0.00)	0 (0.00)	
**Constipation**	13 (12.75)	4 (7.14)	0.277		0 (0.00)	0 (0.00)	
**Pleural effusion**	5 (4.90)	0 (0.00)	0.162		0 (0.00)	0 (0.00)	
**Fatigue**	4 (3.92)	5 (8.93)	0.281		0 (0.00)	0 (0.00)	
**Hand-foot syndrome**	23 (22.55)	5 (8.93)	0.032		2 (1.96)	0 (0.00)	0.539
**Abdominal pain**	58 (56.86)	35 (62.50)	0.491		4 (3.92)	4 (7.14)	0.455

Values are presented as *n* (%).

*P* values were calculated using a two-sided *χ*^2^ test.

ALT: alanine aminotransferase; AST: aspartate aminotransferase.

AE analysis in quadruple therapies is shown in Table 7. No differences in AEs were noted, except the THTI group showed higher leukopenia (*P* < 0.05).

**Table 7:** Treatment-related adverse events between the HTTI group and THTI group

Adverse event	Any grade (cases)				Grade 3–4 (cases)		
	HTTI group (*n* = 61)	THTI group (*n* = 41)	*P* value		HTTI group (*n* = 61)	THTI group (*n* = 41)	*P* value
**Hyperbilirubinemia**	5 (8.2)	4 (9.8)	1.000				
**Hypoalbuminemia**	26 (42.62)	10 (24.4)	0.059				
**Elevated ALT**	7 (11.48)	1 (2.4)	0.139		2 (3.28)	0 (0)	0.514
**Elevated AST**	9 (14.75)	5 (12.2)	0.713		1 (1.64)	0 (0)	1.000
**Increased creatinine**	2 (3.28)	1 (2.4)	1.000				
**Thrombocytopenia**	11 (18.03)	12 (29.3)	0.183		2 (3.28)	2 (4.9)	1.000
**Leukopenia**	13 (21.31)	17 (41.5)	0.029				
**Anemia**	22 (36.07)	13 (31.7)	0.649		2 (3.28)	1 (2.4)	1.000
**Diarrhea**	3 (4.92)	2 (4.9)	1.000				
**Ulcer**	0 (0)	3 (7.3)	0.062				
**Fever**	24 (39.34)	19 (46.3)	0.483				
**Rash**	0 (0)	2 (4.9)	0.159				
**Nausea**	14 (22.95)	11 (26.8)	0.655				
**Constipation**	7 (11.48)	6 (14.6)	0.639				
**Pleural effusion**	4 (6.56)	1 (2.4)	0.646				
**Fatigue**	4 (6.56)	0 (0)	0.147				
**Hand-foot syndrome**	15 (24.59)	8 (19.5)	0.547		1 (1.64)	1 (2.4)	1.000
**Abdominal pain**	35 (57.38)	23 (56.1)	0.898		3 (4.92)	1 (2.4)	0.647

Values are presented as *n* (%)

P values were calculated using a two-sided *χ*^2^ test.

ALT: alanine aminotransferase; AST: aspartate aminotransferase.

Among patients receiving HAIC, three patients (2.94%) discontinued HAIC infusion early due to abdominal pain during four treatment cycles (1.21%), and one patient (0.98%) required oxaliplatin dose reduction during two cycles (0.61%) due to hand-foot syndrome. Across all patients, two individuals (1.27%) required dose reduction of targeted therapy due to rash. No treatment interruptions were observed; no treatment-related mortality occurred.

### Subgroup analyses

Subgroup analysis indicated that, compared to triple therapy, quadruple therapy consistently showed a trend towards better PFS and OS benefits (Supplementary Figure 1, Supplementary Figure 2, available at, http://links.lww.com/JS9/E549). Patients with inferior vena cava tumor thrombus (IVTT) showed particularly significant OS and PFS benefits with quadruple therapy. For patients with total bilirubin above normal, OS benefit was particularly significant with quadruple therapy (*P* interaction < 0.05). Compared to THTI, the HTTI group consistently trended towards better PFS (Supplementary Figure 3, Supplementary Figure 4, available at, http://links.lww.com/JS9/E549). Benefits were more pronounced in patients with lesions confined to the liver and those without bilateral liver involvement (*P* interaction < 0.05).

## Discussion

This study demonstrates that quadruple therapy combining HAIC, TACE, targeted therapy, and immunotherapy significantly improves outcomes for patients with uHCC compared to triple therapy. Importantly, we found that initiating treatment with HAIC followed by TACE yielded superior results compared to TACE-first approach. To maximize broad applicability, inclusion criteria were limited to liver function grading and ECOG performance scores, without limitations distant metastasis, tumor size, distribution, or vascular invasion. According to BCLC guidelines^[5]^,TACE is primarily for BCLC stage B, while systemic therapy is for stage C and selected stage B patients. However, monotherapy with either systemic treatment or TACE shows limited efficacy for advanced HCC. A consensus^[28]^ suggests TACE remains viable for BCLC stage C patients with liver-dominant disease (including major vascular invasion) if Child–Pugh score is <C and performance status is tolerable. Combining TACE with systemic therapy is recognized for enhancing uHCC outcomes. Large multicenter studies, including CHANCE001^[9]^, CHANCE2201^[10]^, LEAP-012^[11]^, and EMERALD-1^[12]^ validated TACE plus targeted therapy and immunotherapy for intermediate-to-advanced HCC. Notably, the CHANCE2201 study emphasized that incorporating TACE into targeted therapy and immunotherapy significantly benefits advanced patients. Therefore, this study designated TACE plus targeted therapy and immunotherapy (triple therapy) as the control.

The FOLFOX regimen^[14,29]^ has significantly enhanced HAIC efficacy in uHCC^[15]^, with promising results in PVTT^[17]^, TACE resistance^[30]^, and challenging scenarios. It also shows advantages in preoperative conversion therapy^[31]^ and postoperative adjuvant therapy^[32]^. As the of HAIC’s clinical importance and distinct mechanism from TACE gain recognition, research on the combination of HAIC and TACE has increased. This combination is particularly beneficial for HCC with PVTT (especially main PVTT), extensive arteriovenous fistulas not effectively embolized by TACE, large tumors, and TACE-resistant disease^[13]^. The advantage of triple therapy is likely linked to the TACE-induced hypoxic microenvironment, upregulating VEGF expression. Antiangiogenic therapy may delay tumor vascular reconstruction post-TACE^[33]^. TACE also triggers the release of tumor antigens and pro-inflammatory cytokines^[34]^, fostering immunogenic cell death, converting “cold tumor” to “hot,” amplifying immunotherapy effectiveness^[35,36]^. Building on this, some studies^[37]^ suggest that chemotherapy induces immunogenic cell death via DNA damage and apoptosis, improving the tumor microenvironment. Antiangiogenic targeted therapies inhibit tumor growth by reducing blood supply while normalizing the tumor vasculature, enhancing the chemotherapy efficacy and reducing resistance. Thus, quadruple therapy may further enhance over synergistic effects over triple therapy. Notably, despite the smaller primary tumor size, fewer tumors, and lower incidences of PVTT and extrahepatic metastasis in the TTI group, quadruple therapy demonstrated superior OS and PFS, supporting its enhanced efficacy for intermediate to advanced HCC. To further validate this finding, we conducted a PSM analysis between the quadruple and triple therapy groups, which confirmed the survival advantage of quadruple therapy after balancing baseline characteristics (Supplementary Table 3, available at, http://links.lww.com/JS9/E549) (Supplementary Figures 5 and 6, available at, http://links.lww.com/JS9/E549).

**Figure 5.**
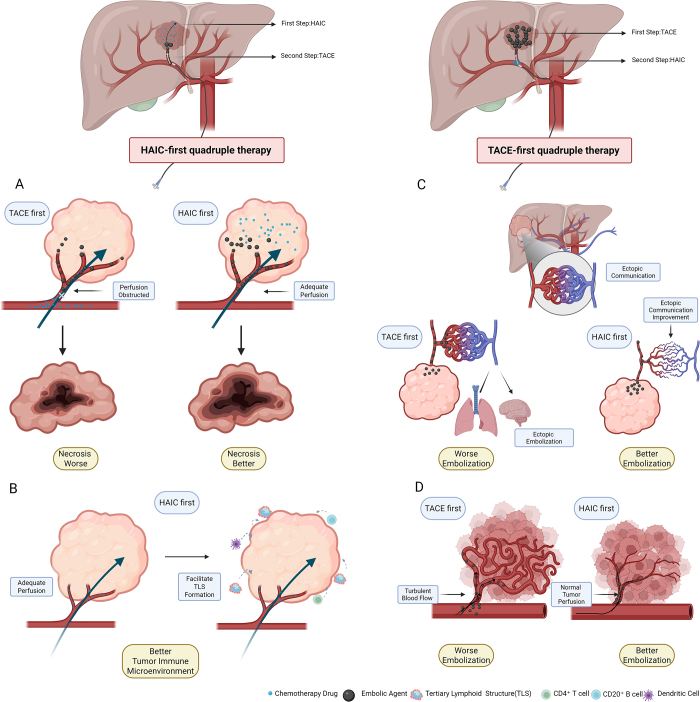
Diagrams of quadruple therapy. (A) Perfusion and necrosis: TACE-first quadruple therapy involves embolizing tumor-feeding arteries before chemotherapy, which may obstruct perfusion and hinder subsequent drug penetration. HAIC-first quadruple therapy delivers chemotherapy first while perfusion remains intact, ensuring effective drug delivery and cytotoxicity, thereby leading to better tumor necrosis. (B) Tumor immune microenvironment: TACE-first impairs drug penetration and limits immune activation. In contrast, HAIC-first maintains adequate perfusion, facilitates the formation of tertiary lymphoid structures and enhances antitumor immune responses. (C) Ectopic embolization: TACE-first may result in ectopic embolization due to ectopic embolization. HAIC-first can reduce tumor volume and improve abnormal vascular communications, thereby reducing the risk of ectopic embolization and enhancing embolization accuracy. (D) Blood flow and embolization quality: TACE-first often faces turbulent or retrograde blood flow, compromising embolization. HAIC-first may help normalize tumor blood flow and support more effective distal embolization.

**Figure 6.**
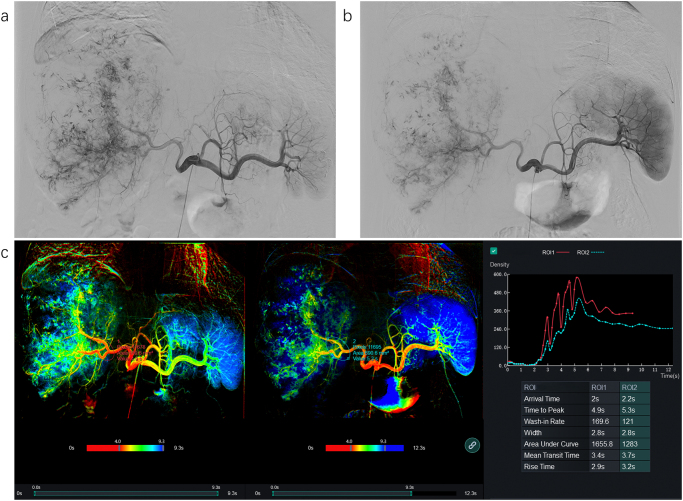
Intraoperative angiography of a patient undergoing HAIC-first quadruple therapy. (A) Hepatic artery angiographic image (pretreatment). (B) Angiography 24 h after FOLFOX infusion (pre-TACE), demonstrating reduced tumor staining, indicative of decreased tumor vascularity following HAIC. (C) Blood flow analysis (pre- and post-angiography). The analysis reveals prolonged time-to-peak, reduced area under the curve, and extended washout time in the target region, indicating a significant reduction in tumor blood supply.

Previous reports of HAIC combined with TACE/TAE treatments^[16,18–21,31,38–48]^ mostly administered TACE first. We propose that that reversing this sequence – HAIC first, then TACE – may be more effective. TACE embolization obstructs tumor blood supply, potentially preventing HAIC drugs from adequately reaching the tumor lesions (Fig. 5A). A previous study has shown that HAIC promotes the formation of tertiary lymphoid structures (TLS) and enhances antitumor immune activity within the tumor microenvironment^[49]^. Therefore, adequate perfusion can facilitate TLS formation and promote improvements in the tumor microenvironment (Fig. 5B). Administering HAIC first allows full drug perfusion and maximized cytotoxic effects before TACE further restricts blood flow. Our previous cases (Fig. 6), where angiography 24 h after FOLFOX showed significant tumor staining reduction, support this. The difference in the follow-up data between the HTTI and THTI groups reflects this pattern: mPFS of the HTTI group was 12.90 months, outperforming the THTI group (10.40 months, *P* = 0.042), HTTI ORR was also slightly higher than THTI. Compared with previous studies evaluating quadruple therapy for uHCC, our study presents several distinguishing features. Most prior research adopted a TACE-first strategy and reported lower ORR and DCR values. For instance, Yuan *et al*^[18]^ reported an ORR of 51.8% and a DCR of 78.4% in patients receiving TACE-HAIC Combination therapy. In contrast, our HAIC-first regimen (HTTI group) achieved an ORR of 81.97% and a DCR of 96.72%, which were noticeably superior to previously reported quadruple therapies^[18,21]^. Although our PFS was slightly shorter than that reported by Yuan *et al*, this may be attributed to the much higher rate of extrahepatic metastasis in our cohort (73.77% vs. 5.7%). When compared with the study by Li *et al*^[19]^, despite our cohort also having a significantly higher rate of extrahepatic metastasis (73.77% vs. 18%), our PFS was markedly longer (12.9 months vs. 8.8 months), further supporting the efficacy of the HAIC-first strategy. Two previous studies also employed an HAIC-first approach followed by TAE^[43,44]^, but their HAIC regimens differed from FOLFOX^[50]^, and the infusion duration (up to 5 days) likely affected patient comfort. A detailed comparison of studies on TACE combined with HAIC is in Table 8.

**Table 8:** Published literature on TACE combined with HAIC

Year of publication	Study and inclusion criteria	Sequence of TACE and HAIC	TACE treatment protocol	HAIC treatment protocol	Targeted therapy	Immunotherapy	Control group	Key findings
2015	Gao *et al*^[16]^, Prospective, non-randomized controlled study: 84 patients with unresectable HCC	TACE-HAIC	cTACE	FOLFOX	No	No	cTACE	ORR: 68.9%
								DCR:86.7%
								mOS: 8.0 m
								mPFS: 4.5 m
2018	Zhu *et al*^[48]^, Retrospective study: 86 patients with portal vein tumor thrombus in HCC	TACE-HAIC	cTACE	Oxaliplatin and Raltitrexed	No	No	NA	ORR: 52.3%
								DCR:75.6%
								mOS: 8.7 m
								mPFS:NA
2020	Guo *et al*^[41]^, Retrospective study: 113 patients with unresectable HCC	TAE-HAIC	TAE	FOLFOX	No	No	cTACE	ORR: 37.14%
								DCR:88.57%
								mOS: NA
								mPFS:7.93 m
2020	Liu *et al*^[46]^, Prospective study: 66 patients with intermediate to advanced HCC	TACE-HAIC	cTACE	FOLFOX	Yes/Sorafenib	No	cTACE	ORR:42.4%
								DCR:87.9%
								mOS: 21.8 m
								mPFS:13.1 m
2021	Huang *et al*^[39]^, Retrospective study: 133 patients with unresectable large HCC (diameter >5.1 cm)	DEB-TACE-HAIC	DEB-TACE	FOLFOX	No	No	DEB-TACE	ORR: 71%
								DCR:94.2%
								mOS: 19 m
								mPFS:9.3 m
2021	Li *et al*^[31]^, Retrospective study: 83 patients with BCLC stage A/B HCC, potentially resectable	TACE-HAIC	cTACE	FOLFOX	No	No	cTACE	ORR: 65.9%
								DCR:92.7%
								mOS: NA
								mPFS:NA
2021	Liu *et al*^[38]^, Retrospective study: 155 patients with advanced HCC and PVTT	TACE-HAIC	cTACE	FOLFOX	No	No	cTACE	ORR: 5.8%
								DCR:53.6%
								mOS: 9.0 m
								mPFS:6.0 m
2021	Chen *et al*^[43]^, Prospective observational cohort study: 32 patients with advanced HCC and PVTT	HAIC (5-day continuous) – TAE	TAE	Cisplatin, Mitomycin-C, and 5-FU	No	No	NA	ORR: 53.1%
								DCR:71.9%
								mOS: 11.9 m
								mPFS:9.5 m
2022	Wu *et al*^[42]^, Retrospective study: 33 patients with advanced HCC and main portal vein thrombus	TACE-HAIC	cTACE	FOLFOX	No	No	cTACE	ORR: 37.5%
								DCR:62.5%
								mOS: 10.0 m
								mPFS:4.78 m
2022	Li *et al*^[19]^, Retrospective study: 101 patients with intermediate to advanced HCC	TACE-HAIC	cTACE	FOLFOX	Yes	Yes	cTACE	ORR: 80%
								DCR:94%
								mOS: 15.6 m
								mPFS:8.8 m
2022	Lin *et al*^[20]^, Retrospective study: 30 patients with uHCC	TAE-HAIC	TAE	FOLFOX	Yes	Yes	NA	ORR: 83.3%
								DCR:100%
								mOS: NA
								mPFS:13.2 m
2022	Li *et al*^[44]^, Retrospective study: 71 patients with advanced HCC and main portal vein thrombus	HAIC (5-day continuous) – TAE	TAE	Cisplatin, Mitomycin-C, and 5-FU	Partial/Sorafenib	No	NA	ORR: 64.8%
								DCR:NA
								mOS: 13.0 m
								mPFS:9.0 m
2023	Chen *et al*^[47]^, Retrospective study: 41 patients with uHCC (diameter > 7 cm)	DEB-TACE/cTACE-HAIC	DEB-TACE/cTACE	FOLFOX	Yes	No	NA	ORR: 56.1%
								DCR:NA
								mOS: NA
								mPFS:8 m
2023	Yuan *et al*^[18]^, Retrospective study: 743 patients with HCC and PVTT	TACE-HAIC	cTACE	FOLFOX	Yes	Yes	cTACE	ORR: 51.8%
								DCR:78.4%
								mOS: NA
								mPFS:14.8 m
2024	Huang *et al*^[21]^, Retrospective study: 123 patients with unresectable HCC	Not specified	cTACE	FOLFOX	Yes	Yes	TACE combined with PD-1 and TKI group	ORR: 20.63%
								DCR:77.78%
								mOS: 21 m
								mPFS:NA
2024	Liu *et al*^[40]^, Retrospective analysis: 167 patients with advanced HCC	TACE-HAIC	DEB-TACE	FOLFOX	No	No	HAIC	ORR: 75.0%
								DCR:93.8%
								mOS:22.4 m
								mPFS: 8.9 m

BCLC: Barcelona Clinic Liver Cancer; cTACE: conventional transcatheter arterial chemoembolization; DCR: disease control rate; DEB-TACE: drug-eluting bead transarterial chemoembolization; FOLFOX: oxaliplatin, 5-fluorouracil, and folinic acid; HAIC: hepatic arterial infusion chemotherapy; m:months; mOS: median overall survival; mPFS: median progression-free survival; ORR: objective response rate; PVTT: portal vein tumor thrombus; TACE: transcatheter arterial chemoembolization; TAE: transcatheter arterial embolization; uHCC: unresectable hepatocellular carcinoma.

**Figure 7.**
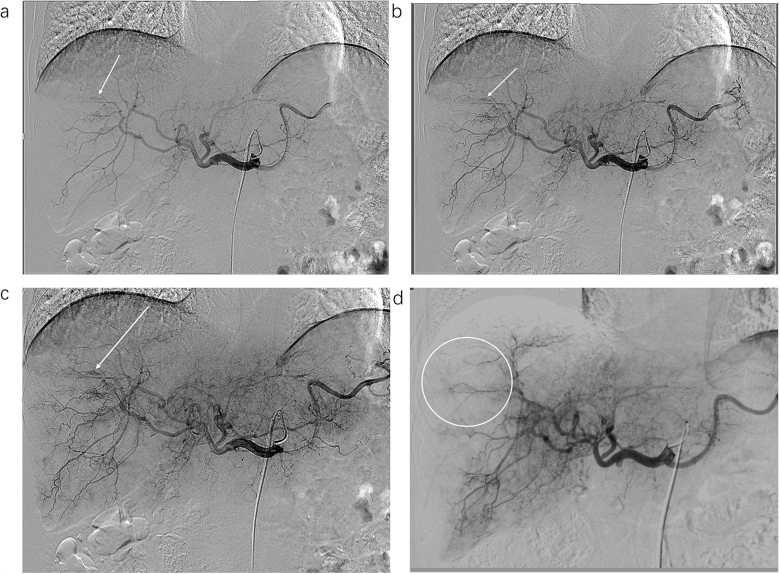
Intraoperative angiography of a patient undergoing HAIC-first quadruple therapy. (a–c) Angiography of the hepatic artery at different phases before HAIC treatment. The arrow indicates a small ectopic communication. (d) Angiography 24 h after FOLFOX. The circular area shows the disappearance of the previously observed ectopic communication.

**Figure 8.**
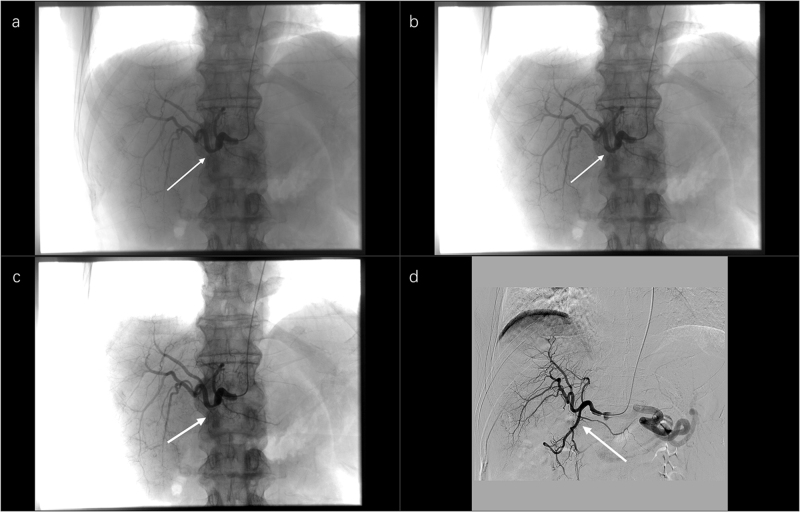
Intraoperative angiographic images before and after HAIC treatment. (a) Hepatic artery angiography before treatment. The arrow indicates nonvisualization of the gastroduodenal artery, which normally branches from the common hepatic artery. (b) Hepatic artery angiography before treatment. The arrow indicates a localized area of reduced density in the central portion of the right hepatic artery, suggesting retrograde blood flow from the gastroduodenal artery. (c) Hepatic artery angiography before treatment. The arrow shows a mild filling defect in the intrahepatic vasculature. (d) One month after HAIC treatment. Angiography demonstrates restored blood supply, with reappearance of the gastroduodenal artery (arrow).

Furthermore, we propose that HAIC before TACE may alleviate abnormal tumor blood flow, restore normal flow, and enhance embolic agent deposition during TACE (Fig. 5C). Hepatic arteriovenous fistula (HAVF), common in advanced liver cancer, contributes to poor post-TACE prognosis^[51]^. Recent studies^[52]^ show that HAIC plus systemic therapy significantly impacts HAVF, with 74.1% achieving complete closure without recanalization. Although we did not observe such pronounced effects, we noted resolution of smaller ectopic communications (Fig. 7), possibly due to HAIC’s tumor-shrinking effect. Thus, HAIC first may reduce ectopic communications or fistulas, lowering the risk of ectopic embolization during TACE and improving efficacy. For severe AVF with significant flow diversion, we adhere to the standard pre-treatment fistula embolization. HAIC may also regulate abnormal arterial blood flow; we observed that HAIC infusion reduced retrograde flow. We hypothesize that HAIC may aid normal tumor perfusion (Fig. 5D), prevent retrograde flow, and facilitate effective distal embolization during TACE (Fig. 8).

Certainly, TACE followed by HAIC has advantages, such as fewer operating room visits, thereby alleviating psychological stress and financial burden. However, as chemotherapy agents, embolic materials, and equipment are consistent regardless of sequence, the primary additional cost for HAIC-first is one extra operating room session. In our cohort this was less than 5% of total medical expenses, a minimal financial burden. While HAIC first may slightly increase procedural waiting time, our center’s experience indicates that patients in both sequences typically have compression devices removed by the third day morning, so overall bed rest and hospital stay are unaffected.

Our multivariate analysis revealed quadruple therapy as an independent protective factor for both OS and PFS. Elevated direct bilirubin was an independent high-risk factor for OS; bilateral liver involvement for PFS. Elevated direct bilirubin is a reported negative prognostic factor in various^[53,54]^. Bilateral liver involvement can impact HAIC efficacy of, as typically only one liver lobe is infused. Our subgroup analysis showed that patients with IVTT and elevated total bilirubin derived significant benefits from the quadruple therapy, possibly due to the excellent therapeutic effect of HAIC on IVTT. Previous studies^[55]^ show HAIC plus targeted therapy significantly extends OS/PFS in IVTT/right atrium thrombus patients compared to targeted therapy alone. Thus, IVTT patients showed significant benefits with quadruple therapy. For patients with elevated total bilirubin, quadruple therapy significantly improved OS, possibly due to combined TACE and HAIC efficacy, which also reduced TACE, minimizing liver function damage. Subgroup PFS analysis in quadruple therapy recipients revealed more benefit for patients with lesions confined to one liver lobe (no distant metastasis or bilateral involvement) benefited more noticeably. This might be because, for bilateral involvement, after main lesion necrosis. TACE on one side and HAIC on the contralateral side is feasible in subsequent treatments, diminishing inter-modality differences. Therefore, for patients with IVTT and elevated total bilirubin, quadruple therapy should be prioritized. For lesions confined to one liver lobe, sequential HAIC-TACE should be considered.

Regarding AEs, hand-foot syndrome, hypoalbuminemia, and thrombocytopenia were slightly higher in the quadruple therapy, while other AEs showed no significant differences. No significant difference in grades 3 and 4 AE was noted. Thrombocytopenia, hypoalbuminemia, and hand-foot syndrome are known HAIC-associated AEs^[56,57]^, with hand-foot syndrome likely oxaliplatin-related^[58]^. Most patients tolerated these AEs; no treatment-related deaths occurred. These findings suggest that despite additional chemotherapy, AEs of quadruple therapy remain manageable.

## Limitations

Our study has several limitations. First, its retrospective, single-center design and limited sample size may impact generalizability. Second, the relatively short follow-up period in the HTTI group poses a limitation. Although significant improvements in PFS and ORR were observed, it remains uncertain whether these early advantages will translate into long-term OS benefits. Continued follow-up and prospective validation are essential to confirm the durability of these outcomes. Third, baseline data were not fully matched; TTI patients had less severe conditions, possibly due to selection bias, as our previous studies often used HAIC combined with TACE for more advanced cases. Subgroup analyses and consistent treatment protocols aimed to mitigate this. Nonetheless, despite greater disease severity in quadruple therapy groups, they demonstrated a clear advantage over triple therapy.

Lack of intraoperative imaging and lipiodol-related data prevented detailed procedural difference analysis (e.g., if HAIC before TACE reduces lipiodol or increase embolization volume). Although preoperative imaging indicated that most primary tumors were unilateral, the necessity for multiple repeat treatments raises questions about contralateral catheter placement after unilateral embolization (Supplementary Figure 4, available at, http://links.lww.com/JS9/E549), potentially reducing outcome differences between the HTTI and THTI. Large-scale, multicenter, prospective, randomized controlled trials are needed to validate HAIC-first efficacy and optimize treatment protocols.

## Conclusions

Our study demonstrates that quadruple therapy, combining HAIC, TACE, targeted therapy, and immunotherapy, is a safe and effective treatment for intermediate-to-advanced HCC. Compared to triple therapy, quadruple therapy may provide superior clinical benefits, even in patients with complex disease features such as vascular invasion or extrahepatic spread.

Importantly, this study provides early evidence that the sequence of locoregional therapies may influence treatment efficacy. Initiating with HAIC before TACE appears to improve drug delivery and tumor response, supporting its consideration as a preferred approach. These findings may help guide clinical decision-making, particularly in tailoring treatment strategies for high-risk or advanced-stage patients.

However, the shorter HTTI follow-up raises uncertainty about the long-term OS impact of observed PFS and ORR advantages. Further prospective studies with extended follow-up are warranted to validate these outcomes and inform practice guidelines.

## Data Availability

The data supporting the findings of this study are available from the corresponding author upon reasonable request.

## Research registration unique identifying number (UIN)

Registry used registration ID: researchregistry11079 Hyperlink: https://www.researchregistry.com/browse-theregistry#home/registrationdetails/67c178f509096a02d62b8032/
